# Reference intervals for plasma amyloid-β, total tau, and phosphorylated tau181 in healthy elderly Chinese individuals without cognitive impairment

**DOI:** 10.1186/s13195-023-01246-1

**Published:** 2023-05-26

**Authors:** Jingshan Chen, Xue Zhao, Wenyan Zhang, Tianxiang Zhang, Siting Wu, Jinghao Shao, Fu-Dong Shi

**Affiliations:** 1 Department of Neurology, Institute of Neuroimmunology, Tianjin Neurological Institute, Tianjin Medical University General Hospital, Tianjin, 300052 China; 2 Division of Neuroimmunology, China National Clinical Research Center for Neurological Diseases, Beijing Tiantan Hospital, Beijing, 100070 China

**Keywords:** Alzheimer’s disease, Biomarkers, Reference intervals, Cognitively normal, Elderly

## Abstract

**Background:**

Plasma amyloid-β (Aβ) peptides and tau proteins are promising biomarkers of Alzheimer’s disease (AD), not only for predicting Aβ and tau pathology but also for differentiating AD from other neurodegenerative diseases. However, reference intervals for plasma biomarkers of AD in healthy elderly Chinese individuals have not yet been established.

**Methods:**

Biomarkers of AD were measured using single-molecule array (Simoa) assays in plasma samples from 193 healthy, cognitively unimpaired Chinese individuals aged 50–89 years. The 95% reference intervals for plasma Aβ42, Aβ40, t-tau, p-tau181, and derived ratios were calculated by using log-transformed parametric methods.

**Results:**

Plasma Aβ42, Aβ40, and p-tau181 levels were positively correlated with age, while the Aβ42/Aβ40 ratio was negatively correlated with age. The 95% reference intervals for plasma Aβ42 and Aβ40 were 2.72–11.09 pg/mL and 61.4–303.9 pg/mL, respectively, and the 95% reference intervals for plasma t-tau and p-tau181 were 0.20–3.12 pg/mL and 0.49–3.29 pg/mL, respectively. The 95% reference intervals for the Aβ42/Aβ40 ratio, p-tau181/t-tau ratio, and p-tau181/Aβ42 ratio were 0.022–0.064, 0.38–6.34, and 0.05–0.55, respectively.

**Conclusion:**

Reference intervals for plasma biomarkers of AD may assist clinicians in making accurate clinical decisions.

**Supplementary Information:**

The online version contains supplementary material available at 10.1186/s13195-023-01246-1.

## Introduction

Alzheimer’s disease (AD) is the most common neurodegenerative disorder worldwide and is also one of the main health problems affecting elderly people [1]. AD is an irreversible and progressive brain disease characterized by memory loss, cognitive deficits, and personality changes. The pathologic hallmarks of AD are the accumulation of amyloid-β (Aβ) protein in the extracellular space and the aggregation of tau-containing neurofibrillary tangles (NFTs) inside cortical neurons [2]. Aβ plaques consist mainly of insoluble Aβ peptides, among which Aβ42 and Aβ40 are the most important isoforms. Aβ42 and Aβ40 have various functions and effects on AD pathology. Aβ42 aggregates are the major components of amyloid plaques in the brains of AD patients, and Aβ40 aggregates are predominantly involved in cerebral amyloid angiopathy [3, 4]. The major components of NFTs are insoluble fibres formed by the self-aggregation of hyperphosphorylated tau protein. Human tau protein, the most abundant microtubule-associated protein in the brain, is mainly located in neurons of the central nervous system neurons and is expressed at low levels in astrocytes and oligodendrocytes [5, 6]. Tau protein acts as a microtubule stabilizer and plays important roles in microtubule assembly, synaptic signal transmission and neural communication. Phosphorylated tau is believed to dissociate from dendrites and form neurofibrillary tangles in the brains of AD patients. Tau protein phosphorylated at threonine 181 (p-tau181) is a tau isoform that is considered a specific marker for AD detection and is more strongly associated with AD than total tau (t-tau) is [7–9].

The National Institute on Aging–Alzheimer’s Association (NIA-AA) research framework specified the importance of Aβ, tau impairment, and neurodegeneration [AT(N)] in the biological definition of AD [10]. The AT(N) scheme is evaluated by testing for Aβ42, t-tau, and p-tau in cerebrospinal fluid (CSF) or by amyloid and tau PET imaging. However, each of these approaches is either relatively invasive or too expensive to perform in primary care clinics. Blood-based detection techniques for these biomarkers have been explored to overcome these drawbacks. However, measuring these biomarkers in blood has historically been a challenge because they are present at lower concentrations in blood than in CSF. Recently, ultrasensitive single-molecule array (Simoa) technology has enabled the reliable quantification of AD biomarkers in the blood of healthy individuals and individuals with neurological diseases; this technology has the potential to be applied in clinical practice [11, 12]. However, reference intervals for plasma AD biomarkers in healthy individuals have not been reported. In this study, we established reference intervals of plasma Aβ42, Aβ40, t-tau, p-tau181, Aβ42/Aβ40 ratio, p-tau181/t-tau ratio, and p-tau181/Aβ42 ratio in healthy elderly Chinese individuals without cognitive impairment and analysed the factors influencing these variables.

## Materials and methods

### Participants

The subjects of this study were community-dwelling elderly people living in Dongli District, Tianjin. Participants with a history of stroke, traumatic brain injury, cognitive impairment, psychiatric disorders, or other neurological diseases were excluded from the study. A total of 193 subjects (97 men and 96 women) aged 50–89 years were enrolled in the study. Participants were divided into four subgroups based on age: 50–59 years (*n* = 56), 60–69 years (*n* = 61), 70–79 years (*n* = 49), and 80–89 years (*n* = 27). Systematic interviews were conducted by general practitioners. The Mini-Mental State Examination (MMSE) was used to evaluate cognitive function. Normal cognition was defined as an MMSE score ≥ 27 for individuals with 7 or more years of education and an MMSE score ≥ 25 for individuals with 1–6 years of education [13]. The demographics and characteristics of the healthy participants are shown in Table 1. All participants or their caregivers signed informed consent before participating in the study. The study was approved by the Ethics Committee of Tianjin Medical University General Hospital (IRB2019-KY-047).

**Table 1:** Characteristics of subjects involved in the study and levels of plasma biomarkers for Alzheimer’s disease

Characteristics	Age groups				
	50–59 (*n* = 56)	60–69 (*n* = 61)	70–79 (*n* = 49)	80–89 (*n* = 27)	Total (*n* = 193)
Age (years)	55.8 ± 2.5	65.0 ± 2.9	73.8 ± 2.8	83.6 ± 2.6	67.3 ± 9.8
Male (*n*, %)	25 (44.6)	30 (49.2)	31 (63.3)	11 (40.7)	97 (50.3)
Education (*n*, %)^*^					
≥ 7 years	44 (78.6)	39 (63.9)	29 (59.2)	5 (18.5)	117 (60.6)
1–6 years	12 (21.4)	22 (36.1)	20 (40.8)	22 (81.5)	76 (39.4)
MMSE	26.9 ± 2.9	26.3 ± 2.6	26.1 ± 1.9	25.5 ± 0.9	26.3 ± 2.6
Haemoglobin (g/L)	147.18 ± 13.42	149.71 ± 13.80	146.64 ± 13.97	141.30 ± 16.19	146.70 ± 14.28
Total cholesterol (mmol/L)	5.74 ± 1.04	5.55 ± 1.23	5.54 ± 0.78	5.42 ± 1.02	5.57 ± 1.03
Triglyceride (mmol/L)	2.01 ± 1.35	1.49 ± 0.77	1.47 ± 0.73	1.46 ± 0.69	1.64 ± 0.99
HDL cholesterol (mmol/L)	1.22 ± 0.26	1.30 ± 0.32	1.27 ± 0.33	1.28 ± 0.29	1.26 ± 0.30
LDL cholesterol (mmol/L)	3.42 ± 0.78	3.41 ± 0.94	3.36 ± 0.66	3.16 ± 0.81	3.36 ± 0.81
Creatinine (μmol/L)^b,c,d,e^	63.01 ± 14.73	64.79 ± 11.46	71.67 ± 12.83	72.27 ± 12.34	68.41 ± 20.29
Glucose (mmol/L)	6.62 ± 1.72	6.50 ± 1.68	6.73 ± 1.92	6.91 ± 2.39	6.65 ± 1.79
Aβ42 (pg/mL)^b,c,d,e^	6.42 ± 1.98	6.50 ± 1.70	7.36 ± 2.58	8.04 ± 1.98	6.91 ± 2.13
Aβ40 (pg/mL)^b,c,d,e,f^	168.57 ± 57.24	161.18 ± 50.86	197.05 ± 62.47	236.06 ± 57.81	182.6 ± 61.9
Aβ42/Aβ40 ratio^c,e^	0.039 (0.034, 0.046)	0.038 (0.034, 0.049)	0.037 (0.032, 0.045)	0.035 (0.031, 0.038)	0.038 (0.033, 0.045)
t-tau (pg/mL)	0.80 (0.53, 1.28)	0.67 (0.44, 1.01)	0.86 (0.52, 1.33)	1.06 (0.57, 1.57)	0.78 (0.52, 1.31)
p-tau181 (pg/mL)^a,b,c^	0.90 (0.77, 1.29)	1.18 (0.96, 1.77)	1.31 (0.97, 1.93)	1.41 (1.11, 2.58)	1.19 (0.88, 1.76)
p-tau181/t-tau ratio	1.14 (0.77, 1.89)	1.82 (1.17, 2.95)	1.63 (0.89, 2.78)	1.40 (0.93, 2.98)	1.58 (0.90, 2.71)
p-tau181/Aβ42 ratio	0.14 (0.10, 0.22)	0.18 (0.14, 0.27)	0.22 (0.11, 0.33)	0.19 (0.15, 0.30)	0.18 (0.12, 0.28)

Data are expressed as the mean±standard deviation or median (interquartile range).* P* values: a = 50–59 vs 60–69 ≤ 0.05, b = 50–59 vs 70–79 ≤ 0.05, c = 50–59 vs 80–89 ≤ 0.05, d = 60–69 vs 70–79 ≤ 0.001, e = 60–69 vs 80–89 ≤ 0.05, f = 70–79 vs 80–89 ≤ 0.05. *P ≤ 0.05

*Abbreviations: MMSE* Mini-Mental State Examination, *HDL* High-density lipoprotein, *LDL* Low-density lipoprotein, *Aβ* Amyloid-beta protein, *t-tau* Total tau, *p-tau181* Tau phosphorylated at threonine 181

### Processing of samples

Blood samples were drawn by the laboratory technician. Fasting venous blood was drawn from all subjects between 8 am and 10 am and collected in tubes containing ethylenediaminetetraacetic acid (EDTA). Samples were then centrifuged at 3000 × *g* for 10 min at 4°C to obtain plasma within 2 h of collection. The plasma was stored at −80°C until biochemical analysis. All measurements were performed on the Simoa HD-X analyser platform (Quanterix, Lexington, MA). Plasma Aβ42, Aβ40, and t-tau levels were measured using the Quanterix Simoa Neurology 3-Plex A Advantage kit (Lot 503205), and p-tau181 levels were measured using the p-tau181 Advantage V2 kit (Lot 502793), all according to the manufacturer’s instructions and standard procedures. The lower limits of detection of the Aβ42, Aβ40, t-tau, and p-tau181 assays were 0.045, 0.196, 0.019, and 0.028 pg/mL, whereas the lower levels of quantification were 0.142, 0.675, 0.063, and 0.338 pg/mL, respectively. The coefficients of variation for Aβ42, Aβ40, t-tau, and p-tau181 were 5.0%, 9.7%, 6.2%, and 4.7%, respectively. Samples were diluted 4× and tested in duplicate by the automatic HD-X analyser. Two quality control samples were run on each plate for each analyte.

### Statistical analysis

The normality of distributions was tested using the Kolmogorov–Smirnov test. Data that followed a normal distribution are presented as the mean ± standard deviation (SD), and data that did not follow a normal distribution are expressed as the median and interquartile range (IQR). Categorical variables are expressed as numbers and percentages. Chi-square test was used to analyse differences in frequencies between groups. One-way ANOVA followed by Fisher’s LSD post hoc test were applied to compare demographic characteristics and plasma AD biomarkers (Aβ42, Aβ40, t-tau, p-tau181, and their ratios) between age groups. Student’s *t* test was applied to compare demographic characteristics and plasma AD biomarkers between men and women. Multiple linear regression was used to explore the linear relationship between each plasma AD biomarker and age or creatinine levels. The reference intervals for the age groups were calculated according to the Clinical and Laboratory Standards Institute (CLSI) guidelines [14]. The lower and upper limits are represented as the mean ± 1.96×SD. Non-normally distributed data were logarithmically transformed before all analysis. Statistical analysis was performed by using SPSS version 26.0 (IBM Corporation, Armonk, NY, USA) and GraphPad Prism version 8. All tests were two-sided, and the significance threshold was set at *P* < 0.05.

## Results

**Fig. 1:**
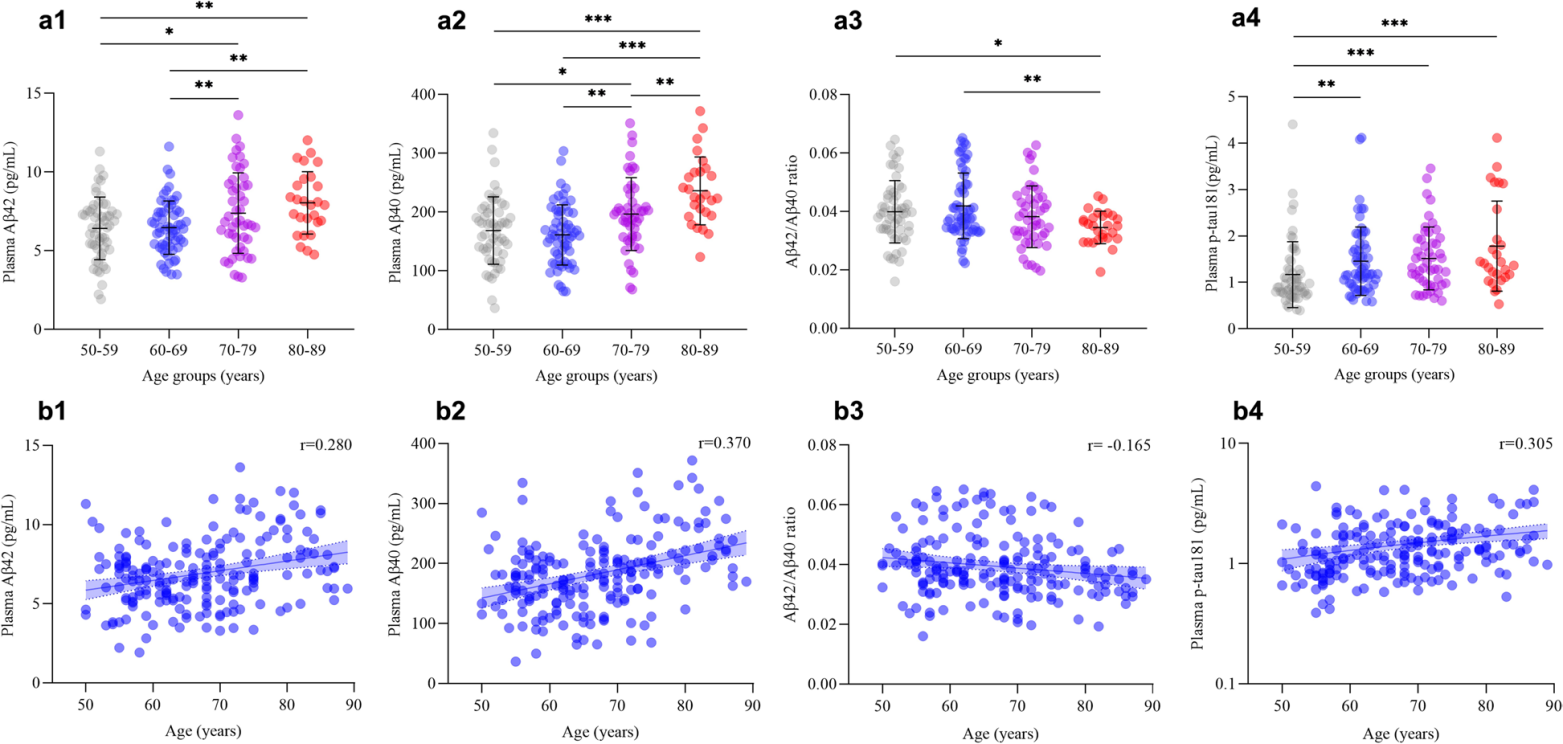
Comparison of plasma levels of amyloid-β 42, amyloid-β 40, amyloid-β 42 to amyloid-β 40 ratio, and phosphorylated tau181 among age groups. **a** (1–4) Interleaved scatter plots show plasma amyloid-β 42, amyloid-β 40, amyloid-β 42 to amyloid-β 40 ratio, and phosphorylated tau181 levels in different age groups. **b** (1–4) Linear regression plots show the correlations between age and plasma amyloid-β 42, amyloid-β 40, amyloid-β 42 to amyloid-β 40 ratio, and phosphorylated tau181 levels. *P ≤ 0.05, **P ≤ 0.01, ***P ≤ 0.001. Abbreviations: Aβ, amyloid-beta protein; t-tau, total tau; p-tau181, tau phosphorylated at threonine 181

Demographic and neuropsychological characteristics, clinical examinations, and levels of plasma biomarkers for AD are presented in Table 1. The mean age of the participants was 67.3 ± 9.8 years, and there was no significant difference in sex between groups. The education level was significantly lower in participants aged 80–89 years than in participants aged 50–59 years, 60–69 years, or 70–79 years. There were no significant differences between the age groups in MMSE scores, or other clinical examinations. The plasma Aβ42 concentration of the participants was 6.91 ± 2.13 pg/mL, and the plasma Aβ40 concentration was 182.6 ± 61.9 pg/mL. The plasma t-tau concentration was 0.78 (IQR, 0.52–1.31) pg/mL, and the plasma p-tau181 concentration was 1.19 (IQR, 0.88–1.76) pg/mL. The levels of plasma Aβ42, Aβ40, Aβ42/Aβ40 ratio, and p-tau181 were significantly different among the four age groups. Plasma Aβ42 concentrations were significantly higher in participants aged 70–79 years (7.36 ± 2.58 pg/mL) or 80–89 years (8.04 ± 1.98 pg/mL) than in participants aged 50–59 years (6.42 ± 1.98 pg/mL) or 60–69 years (6.50 ± 1.70 pg/mL). Plasma Aβ40 concentrations were significantly higher in participants aged 80–89 years (236.06 ± 57.81 pg/mL) than in participants aged 70–79 years (197.05 ± 62.47 pg/mL) and were higher in both of those groups than in participants aged 50–59 years (168.57 ± 57.24 pg/mL) or 60–69 years (161.18 ± 50.86 pg/mL). Aβ42/Aβ40 ratio were significantly lower in participants aged 80–89 years [0.035 (IQR, 0.031–0.038)] than in participants aged 50–59 years [0.039 (IQR, 0.034–0.046)] or 60–69 years [0.038 (IQR, 0.034–0.049)]. The p-tau181 levels in plasma were significantly lower in the 50- to 59-year-old group [0.90 (IQR, 0.77–1.29) pg/mL] than in the 60- to 69-year-old [1.18 (IQR, 0.96–1.77) pg/mL], 70- to 79-year-old [1.31 (IQR, 0.97–1.93) pg/mL], and 80- to 89-year-old groups [1.41 (IQR, 1.11–2.58) pg/mL] (Fig. 1a (1–4)).

We also explored how sex affected plasma levels of AD biomarkers by evaluating the differences between men and women (Table S1). There was no significant difference in age between men and women. There were also no significant differences between men and women in MMSE scores, education levels, or clinical examinations. There were no significant differences between men and women in the level of plasma Aβ42 or Aβ40 or in the Aβ42/Aβ40 ratio. We observed that the level of plasma p-tau181 in men [1.37 (IQR, 1.02–1.94) pg/mL] was significantly higher than that in women [1.10 (IQR, 0.80–1.60)]. Men also had a significantly higher p-tau181/t-tau ratio [1.81 (1.02, 2.96)] and p-tau181/Aβ42 ratio [0.21 (0.14, 0.30)] than women [1.34 (0.88, 2.25) and 0.15 (0.10, 0.26), respectively]. There was no difference in plasma p-tau181 levels between men and women in the 50- to 59-year-old, 60- to 69-year-old, or 70- to 79-year-old age group.

The unadjusted linear regression model showed that plasma Aβ42, Aβ40, p-tau181, and p-tau181/t-tau ratio were positively correlated with age, while the Aβ42/Aβ40 ratio was negatively correlated with age. After adjusting for gender, education year, and creatinine levels, plasma Aβ42 (*r* = 0.280, *P* < 0.001), Aβ40 (*r* = 0.370, *P* < 0.001), and p-tau181 (*r* = 0.305, *P* < 0.001) were positively correlated with age, while the Aβ42/Aβ40 ratio (*r* = −0.165, *P* = 0.023) was negatively correlated with age (Table 2 and Fig. 1b (1–4)). There was no correlation between p-tau181/t-tau ratio and age.

**Table 2:** Correlations of plasma biomarkers of Alzheimer's disease with subject age

Plasma biomarkers	Unadjusted		Adjusted	
	*r*	*P* value	*r*	*P* value
Aβ42	0.288	< 0.001	0.280	< 0.001
Aβ40	0.372	< 0.001	0.370	< 0.001
Aβ42/Aβ40 ratio	−0.204	0.014	-0.165	0.023
t-tau	0.027	0.746	0.053	0.462
p-tau181	0.292	< 0.001	0.305	< 0.001
p-tau181/t-tau ratio	0.156	0.033	0.141	0.053
p-tau181/Aβ42 ratio	0.145	0.081	0.084	0.250

The plasma biomarkers concentrations were log transformed. The model was adjusted for gender, education year, and creatinine

*Abbreviations:* Aβ Amyloid-beta protein, *t-tau* Total tau, *p-tau181* Tau phosphorylated at threonine 181

The unadjusted linear regression model showed that plasma Aβ42, Aβ40, and p-tau181 were positively correlated with creatinine levels. After adjusting for age, gender, and education year, plasma Aβ40 (*r* = 0.159, *P* = 0.036) was positively correlated with creatinine levels, while Aβ42 and p-tau181 were no longer correlated with creatinine levels (Table S2).

Reference intervals for plasma biomarkers of Alzheimer’s disease in healthy elderly Chinese are as follows: Aβ42, 2.72–11.09 pg/mL; Aβ40, 61.4–303.9 pg/mL; Aβ42/Aβ40 ratio, 0.022–0.064; t-tau, 0.20–3.12 pg/mL; p-tau181, 0.49–3.29 pg/mL; p-tau181/t-tau ratio, 0.38–6.34; p-tau181/Aβ42 ratio, 0.05–0.55 (Table 3).

**Table 3:** Reference intervals for plasma biomarkers of Alzheimer’s disease across age groups

Plasma biomarkers	Age groups (years)				
	50–59 (*n* = 56)	60–69 (*n* = 61)	70–79 (*n* = 49)	80–89 (*n* = 27)	Total (*n* = 193)
Aβ42 (pg/mL)	2.53–10.31	3.17–9.82	2.31–12.4	4.16–11.93	2.72–11.09
Aβ40 (pg/mL)	56.5–280.8	61.5–260.9	74.6–319.5	122.8–349.4	61.4–303.9
Aβ42/Aβ40 ratio	0.019–0.064	0.023–0.065	0.020–0.062	0.024–0.045	0.022–0.064
t-tau (pg/mL)	0.19–4.68	0.15–3.95	0.24–2.72	0.20–2.65	0.20–3.12
p-tau181 (pg/mL)	0.39–2.67	0.55–3.15	0.59–3.26	0.57–4.29	0.49–3.29
p-tau181/t-tau ratio	0.32–4.93	0.56–6.16	0.36–6.57	0.31–8.63	0.38–6.34
p-tau181/Aβ42 ratio	0.05–0.52	0.07–0.63	0.06–0.70	0.07–0.61	0.05–0.55

Reference intervals are established on the log-transformed variables and transforming back by taking antilogarithms

*Abbreviations: Aβ* Amyloid-beta protein, *t-tau* Total tau, *p-tau181* Tau phosphorylated at threonine 181

## Discussion

Medical reference intervals are ranges of physiological measurements in healthy individuals who are mainly used for descriptive purposes and are the basis for comparing a set of test results for patients. Values outside the reference intervals are not necessarily pathological. Reference intervals are ideally defined on apparently healthy individuals and should be distinguished from clinical decision cut-offs that are derived from known diseased patients [15]. A cut-off value is a fixed value that distinguishes suspected patients from healthy individuals and therefore allows an interpretation of test results. Here, we provide a detailed description of the changes in plasma Aβ, t-tau, and p-tau181 in healthy elderly Chinese individuals without cognitive impairment using ultrasensitive Simoa assays. To the best of our knowledge, this is the first study to establish reference intervals for these plasma AD biomarkers in healthy elderly individuals. Reference intervals of biomarkers are essential for clinical laboratory test interpretation and disease diagnosis [16]. The publicly available reference intervals help to update reference data, for example, extension of age ranges and additional ethnic groups. The reference intervals we report provide more evidence for the possible future use of AD biomarkers in clinical practice.

In this study, we found that age is the main factor influencing plasma AD biomarkers. Plasma levels of Aβ42, Aβ40, and p-tau181 were positively associated with age. Mielke et al. [17] reported that plasma p-tau181 increased with age starting between the ages of 65 and 70 years, but the increase was greatest among those people with elevated brain amyloid content. Mengel et al. [18] reported that plasma Aβ42 levels decreased with age in patients with Down syndrome, which is contrary to our findings. However, most of the patients in that study were under 50 years old, whereas the participants in our study were all over 50 years old. Therefore, it remains uncertain how plasma amyloid changes with age. In this study, we established age-specific reference intervals for plasma biomarkers. We found that age was the major influential factor of Alzheimer's disease plasma biomarkers in the healthy elderly. Sex is another influential factor. Plasma p-tau181 levels are higher in men than in women, but the underlying physiological mechanism is still unclear.

Recently, plasma biomarker ratios have shown good performance in the diagnosis and prognosis of AD. Among these ratios, the plasma Aβ42/Aβ40 ratio correlates with the CSF Aβ42/Aβ40 ratio and Aβ-PET and can identify individuals with abnormal brain Aβ burden or individuals at high risk for future conversion to Aβ-PET positivity with relatively high accuracy [19–21]. The plasma p-tau181/Aβ42 ratio performs well in identifying AD with elevated brain amyloid content in populations with concomitant cerebrovascular disease [22]. P-tau181 accounts for approximately 14% of t-tau as measured by the immunomagnetic reduction method in healthy individuals [23]. However, in this study, the plasma p-tau181 and t-tau concentrations detected by Simoa were not consistent with previous studies. We found that the plasma p-tau181 concentrations were higher than the t-tau concentrations in healthy elderly. Although plasma AD biomarker ratios have not yet been used as diagnostic methods for AD, the reference intervals herein still provide evidence to support the future use of these ratios in clinical practice.

Another advantage of plasma AD biomarker ratios over individual biomarkers is that the ratios are less affected by comorbidities [24]. Multiple common comorbidities, including chronic kidney disease (CKD), myocardial infarction (MI), and stroke, have been reported to be associated with elevated plasma AD biomarkers [17, 25]. Therefore, participants with these comorbidities should be excluded during the establishment of normal reference intervals. In our study, MI and stroke were excluded in all participants, and the subjects had normal serum creatinine levels (57–97 μmol/L) as well. However, the only kidney biomarker we measured was serum creatinine, which does not definitively exclude CKD; the lack of adequate CKD markers may be one of the limitations of this study. In addition, comorbidities should be fully considered in the future use of biomarkers for the clinical screening, diagnosis, or prognosis of AD. We used the MMSE to evaluate the cognitive function of the participants. MMSE performance is strongly affected by education levels, with a greater length of education being associated with better MMSE performance. In the present study, participants were grouped into two tiers of education (1–6 years and 7 or more years), and different MMSE cut-offs were used to define normal cognitive function in these two subsets of our sample. In addition, MMSE performance is slightly affected by age, gender, and place of residence [13]. However, all participants in our study were from adjacent communities; therefore, we did not consider it necessary to adjust for these factors. Our study was conducted in a relatively small population and was not validated in other datasets. In addition, there are differences in plasma marker levels for Alzheimer’s disease measured by different Simoa assays. Therefore, generalizability of the results to other populations might therefore be limited.

In summary, the reference intervals of AD biomarkers need to be further verified in people from different regions and races, and the sample size still needs to be expanded as well. The preliminary estimation of AD biomarker distributions in healthy elderly people will help to determine the appropriate cut-off values of AD biomarkers and provide a reliable basis for the diagnosis of neurodegenerative diseases in China.

## Supplementary Information

**Additional file 1: Table S1.** Characteristics of subjects grouped by sex and plasma biomarker levels of Alzheimer's disease. **Table S2.** Correlations of plasma biomarkers of Alzheimer's disease with subject creatinine levels.

## Data Availability

The datasets used and/or analysed during the current study are available from the corresponding author on reasonable request.

## Abbreviations

Aβ: Amyloid-β
AD: Alzheimer’s disease
Simoa: Single-molecule array
NFTs: Neurofibrillary tangles
p-tau181: Tau protein phosphorylated at threonine 181
t-tau: Total tau
NIA-AA: National Institute on Aging–Alzheimer’s Association
AT(N): Aβ, tau impairment, and neurodegeneration
CSF: Cerebrospinal fluid
MMSE: Mini-Mental State Examination
EDTA: Ethylenediaminetetraacetic acid
SD: Standard deviation
IQR: Interquartile range
CLSI: Clinical and Laboratory Standards Institute
CKD: Chronic kidney disease
MI: Myocardial infarction
