# Supplementary material for: Reference intervals for plasma amyloid-β, total tau, and phosphorylated tau181 in healthy elderly Chinese individuals without cognitive impairment
Source: Alzheimers Res Ther. 2023 May 26;15:100. doi: 10.1186/s13195-023-01246-1 (PMC10214719; doi:10.1186/s13195-023-01246-1)
Supplement: Supplementary file 1 — Additional file 1: Table S1. Characteristics of subjects grouped by sex and plasma biomarker levels of Alzheimer's disease. Table S2. Correlations of plasma biomarkers of Alzheimer's disease with subject creatinine levels. [file 13195_2023_1246_MOESM1_ESM.docx]

Table S1. Characteristics of subjects grouped by sex and plasma biomarker levels of Alzheimer's disease

| Characteristics | Male (n = 97) | Female (n = 96) | Total (n = 193) |
| --- | --- | --- | --- |
| Age (years) | 67.5 ± 9.1 | 67.1 ± 10.4 | 67.3 ± 9.8 |
| Education (n, %) |  |  |  |
| >7 years | 63 (64.9) | 54 (56.3) | 117 (60.6) |
| 1-6 years | 34 (35.1) | 42 (43.8) | 76 (39.4) |
| MMSE | 26.5 ± 2.1 | 26.1 ± 3.0 | 26.3 ± 2.6 |
| Haemoglobin (g/L) | 153.64 ± 13.20 | 139.66 ± 11.71 | 146.70 ± 14.28 |
| Total cholesterol (mmol/L) | 5.36 ± 0.96 | 5.79 ± 1.07 | 5.57 ± 1.03 |
| Triglyceride (mmol/L) | 1.61 ± 1.16 | 1.67 ± 0.79 | 1.64 ± 0.99 |
| HDL cholesterol (mmol/L) | 1.20 ± 0.28 | 1.33 ± 0.31 | 1.26 ± 0.30 |
| LDL cholesterol (mmol/L) | 3.25 ± 0.75 | 3.46 ± 0.86 | 3.36 ± 0.81 |
| Creatinine (μmol/L) | 77.78 ± 23.51 | 58.92 ± 9.63 | 68.41 ± 20.29 |
| Glucose (mmol/L) | 6.63 ± 1.57 | 6.68 ± 1.99 | 6.65 ± 1.79 |
| Aβ42 (pg/mL) | 6.77 ± 2.07 | 7.04 ± 2.19 | 6.91 ± 2.13 |
| Aβ40 (pg/mL) | 184.4 ± 64.3 | 180.8 ± 59.6 | 182.6 ± 61.9 |
| Aβ42/Aβ40 ratio | 0.037 (0.031, 0.043) | 0.039 (0.033, 0.046) | 0.038 (0.033, 0.045) |
| t-tau (pg/mL) | 0.76 (0.51, 1.33) | 0.82 (0.54, 1.31) | 0.78 (0.52, 1.31) |
| p-tau181 (pg/mL) ^*^ | 1.37 (1.02, 1.94) | 1.10 (0.80, 1.60) | 1.19 (0.88, 1.76) |
| p-tau181/t-tau ratio^*^ | 1.81 (1.02, 2.96) | 1.34 (0.88, 2.25) | 1.58 (0.90, 2.71) |
| p-tau181/Aβ42 ratio^*^ | 0.21 (0.14, 0.30) | 0.15 (0.10, 0.26) | 0.18 (0.12, 0.28) |

Data are expressed as the mean ± standard deviation or median (interquartile range). **P* ≤ 0.05. Abbreviations: MMSE, Mini-Mental State Examination; HDL, high-density lipoprotein; LDL, low-density lipoprotein; Aβ, amyloid-beta protein; t-tau, total tau; p-tau181, tau phosphorylated at threonine 181.

Table S2. Correlations of plasma biomarkers of Alzheimer's disease with subject creatinine levels

| Plasma biomarkers | Unadjusted | | Adjusted | |
| --- | --- | --- | --- | --- |
|  | r | *P* value | r | *P* value |
| Aβ42 | 0.202 | **0.011** | 0.139 | 0.083 |
| Aβ40 | 0.262 | **< 0.001** | 0.159 | **0.036** |
| Aβ42/Aβ40 ratio | -0.067 | 0.405 | -0.009 | 0.908 |
| t-tau | 0.054 | 0.497 | 0.080 | 0.338 |
| p-tau181 | 0.171 | **0.031** | 0.128 | 0.102 |
| p-tau181/t-tau ratio | 0.052 | 0.518 | -0.008 | 0.919 |
| p-tau181/Aβ42 ratio | 0.059 | 0.460 | 0.019 | 0.818 |

The plasma biomarkers concentrations were log transformed. The model was adjusted for age, gender, education year and creatinine. Abbreviations: Aβ, amyloid-beta protein; t-tau, total tau; p-tau181, tau phosphorylated at threonine 181.
